# Dental Economics—One View Point

**Published:** 1910-11-15

**Authors:** C. Edson Abbott


### DENTAL ECONOMICS—ONE VIEW POINT.

BY C. EDSON ABBOTT, D.D.S.

In sympathy with the times, and with various other editors who have discussed this topic, the writer presents his views of man's dental needs and the goods and services which satisfy them.

Some editors discuss dentistry from the standpoint of art for art's sake. Others study how to add to good work such additional utilities as superfine furniture, social prestige, superior attendance, stationery, location, harmonious atmosphere, etc., in order to exact relatively higher fees based on the higher cost of such service, the limited amount of such service which may be produced, and the desire of the wealthy for the distinctive and rare. High fees and higher fees is usually the ultimate appeal of most writers on the business side of dentistry. The narrowness of the first class of writers, who do seem to realize the vast need of the world for our services, which could be multiplied in volume by a fraction of the diligent study which the business man applies to his production and distribution of goods for mankind's use, and the lack of historical perspective on the part of the second class, who fail to realize that machinery and system have added to mankind's happiness because they have created vast quantities of useful goods for man's use at a fair price which he is able to pay, do not obscure my vision.

I see the dentist as a producer of economic goods, whose utilities satisfy a demand from consumers. To him the observed laws of economics apply. A systematic study of dental economics in dental schools, and in dental societies would result in his greater service to humanity, through lessening of waste and emphasizing the important. The service of mankind and the happiness of the dentist demand alike that the great mass of dentists serving the people with necessary dental operations, shall have adequate instruction in the distribution of these services, as well as the production. For such a course the writer would suggest lectures in:

General Economics.

History of Industry.

Dental Economics.

The Dentist, his Equipment, and his Operations.

Bookkeeping.

Accounting.

Increasing Production.

Avoiding Waste.

Buying.

Collections.

Investment.

Such knowledge will raise the average dentist to wider usefulness, financial independence and a broader look on all life's problems. "The happiness of the great mass of mankind is the goal of the philosopher," says Prof. Charles Eliot. "The rich and famous are too few to affect appreciably the sum of human happiness." If dental work is worth while then increasing its amount by application of business methods is surely worth while to the public.

A few brief illustrations will show how consideration of the last eight divisions suggested help the average dentist.

Machinery and equipment cost the first investment, operation and repair, and replacement when worn or obsolete. Not subject to wage expense, fatigue, sickness or sudden resignation. The most complete equipment will pay any dentist many times more than any safe investment.

Standard goods should be bought in large quantities. Five humdred dollars invested here will pay 100 per cent. per year, by being turned over several times.

Labor is the greatest expense of any practice by far. His own is the highest wage, and the care of his office, his book-keeping, his correspondence, his errands, his banking, his telephoning, etc., are more economically done by an assistant. All vulcanizing and finishing and most laboratory work, according to system and individuality of the dentist should be turned over to a good assistant or laboratory, unless the dentist prefers to specialize in prosthesis. Wages is the reward paid to labor for its share in industry. The dentist should earn not only wages of production, but wages of management and wages of distribution.

The dentist should do what he excels in, and let assistants do the rest. This lessens the wage expense of each operation and increases the net profit. By thus saving time and strength the dentist is enabled to give patients attention on short notice, to accomplish more work in the time the patient spends in the office, and to consider those niceties which attract patients.

Arrangement of appointments requires that when possible the difficult operations should be done in the early forenoon, when fatigue and interruption is less. Doubtful appointments should be preceded by sure ones, to permit overlapping. It is safe to reserve only short periods for patients careless of appointments.

Prompt, accurate bookkeeping. This records all work and fees, favors promp settlement of accounts, explains misunderstandings, shows the dentist weak spots. Accounting and analysis of expenses, losses and profits, furnish the data for scientific analysis of the business side of the practice. The up-to-date business man pities the old-timer, yet the latter is ahead of most dentists in business methods.

The proper grouping of operations requires that treatments be commenced at the first sitting, that extractions occur at the end of a sitting, that operations in adjacent teeth be done together, or at several days' interval, that tedious operations be not done at the first sitting, that the final finishing of several similar fillings be done at one sitting, that operations be not carried to the point of undue fatigue or pain, etc.

Credit and Collections. A man who can attract more work than he can do may well accept as patients only those of known good credit. Otherwise he may deem it wiser to do the largest possible gross business and collect it well. To have no system either in credits or collections results in a large loss.

Investment. Saving a little each year, investing in safe properties at reasonable interest, when young, protecting the family with insurance, avoiding extravagant living and get rich quick schemes makes for comfort.

Charities If the dentist is forehanded in the above matters he can afford to give time to free charity work for children recommended by some organization or school or other officials, and to educational work in public lectures. This is better than involuntary charity to deadbeats, which absorbs the strength of many dentists careless in business methods.

We have here offered food for thought for the average dentist serving average people. Is it not worthy of consideration?—*The Journal.*

